# Organizational humanization: definition and measurement

**DOI:** 10.3389/fpsyg.2026.1906924

**Published:** 2026-10-20

**Authors:** Kevin Derek Stroop

**Affiliations:** Department of Psychology, School of Behavioral Sciences, Liberty University, Lynchburg, VA, United States

**Keywords:** autonomy, dehumanization, dignity, engagement, humanization, individuality, measurement, social capital

## Abstract

Research on humanization is fragmented, generally focuses on dehumanization, and prior to this study no validated measure of organizational humanization existed. To address this gap, I defined organizational humanization and evaluated the Organizational Humanization Scale (OHS), a nine-item measure of perceived organizational humanization. The OHS integrates autonomy, individuality, and dignity as related dimensions and uses adapted items from established measures of work autonomy, work group inclusion, and workplace dignity to preserve continuity with the content of those measures. Using a cross-sectional survey of full-time employed adults in the United States (*N* = 441), I evaluated the OHS's internal consistency reliability and evidence of structural, convergent, internal discriminant, and nomological validity. Results supported a second-order factor structure representing a humanization general factor reflected by autonomy, individuality, and dignity subscales. The OHS showed positive associations with workplace social capital and work engagement. Comparisons with established measures of autonomy, inclusion, and dignity on which the OHS was based showed similar associations despite the OHS consisting of fewer items. These findings provide initial support for the OHS as a concise measure for integrating autonomy, individuality, and dignity within organizational humanization research and applied assessment.

## Introduction

Dehumanization of people within an organization erodes the foundation on which the organization is built. Yet reducing dehumanization is not the same as humanization. If dehumanization is a process of denial, humanization must be understood as a process of affirmation. To date, however, organizational research lacks an integrated definition or measure of humanization. This study addresses that gap by integrating autonomy, individuality, and dignity into a definition and measure of organizational humanization.

Two major world events have highlighted the consequences of a lack of focus on people within organizations: the COVID-19 pandemic and the emergence of artificial intelligence (AI). The pandemic exposed how unprepared organizations were for disruption and called for attention to business continuity and organizational resilience (Payne et al., 2021; Su and Junge, 2023). Rapid attempts to integrate AI in businesses have also raised concern that digital transformations may dehumanize employees rather than simply augment their work (Gibson et al., 2025). The commonality in both world events, and similar smaller-scale contexts, is not the event itself, but the lack of emphasis on people within organizations when responding to disruptions or change.

During the pandemic, widespread job loss and slow organizational recovery reduced employee engagement due to concerns with job security and work arrangements (Ployhart et al., 2021; Hur, 2022). Studies looking back at organizational responses to disruptions identified human factors including social capital as relevant to organizational resilience (DiBella et al., 2023). In some contexts, researchers found that social capital contributed to resilience more than other types of capital because the relationships can improve access to resources during a crisis (Mzid et al., 2019). Despite this, research increasingly emphasized digitalization and related factors as important directions for organizational resilience (Paeffgen et al., 2024), rather than emphasizing human factors. This creates tension between addressing organizational concerns and supporting people within those organizations, which suggests that some responses to the pandemic, including AI, may have addressed resilience but created new risks for how people experience treatment in organizations.

Despite these events drawing attention to the treatment of people within organizations, current research has remained far more focused on dehumanization than humanization. Dehumanization research has largely built on the dual model of animalistic and mechanistic dehumanization (Haslam, 2006). Since then, researchers have continued to develop the construct, including subtle and blatant forms, a growing range of methods and consequences, and interventions to reduce dehumanization (Haslam and Loughnan, 2014; Kteily and Landry, 2022), without a corresponding body of research on humanization.

Research should not assume that humanization and dehumanization are opposite ends of a single continuum. In dehumanization, people recognize the target as human in some manner while regarding the target as less than fully human in essence (Smith, 2016). This is a paradox of dehumanization. In the process of dehumanizing a person, one necessarily first recognizes their humanness to be denied in the first place. While lower dehumanization may indicate fewer instances of denial, it does not indicate any particular instance of affirmation. Even if denial were absent, the result could be neutrality or simply an absence of attention. For example, not treating someone disrespectfully does not establish that the person's contributions have been recognized and respected. Humanization therefore has to be assessed directly rather than inferred from the absence of dehumanization.

In organizations, particularly in businesses, people are often referenced through the functions they perform rather than through the person as a whole or their humanness. Before the pandemic, researchers linked strategic human resource management (HRM) to resilience through policies, practices, and desired employee contributions, including collaboration, trust, and self-management (Lengnick-Hall et al., 2011). After the pandemic, HRM research continued to link resilience with organizational processes, including competences, recruitment, training, performance management, and the work environment (Macchi Silva and Duarte Ribeiro, 2022; Georgescu et al., 2024). These approaches recognize that people are central to resilience, but they still divide the person into fragmented components. If organizations treat people in these ways, they risk a reductionist form of dehumanizing treatment because the person is no longer treated as a whole person (Domingo and Melé, 2022). In effect, the organization does not affirm humanness despite recognizing people as central to the organization's interests.

This risk extends into research that emphasizes digitalization, partnerships, and learning as important topics, while other topics, including preparedness for disruptions, receive less attention (Paeffgen et al., 2024). Yet research links digital transformation to dehumanization, with calls to rehumanize work as automation and AI reshape employee experiences (Gibson et al., 2025; Grabowska et al., 2022). AI use can create dehumanization risks when users feel treated as objects by AI, when AI use weakens perceptions of control or autonomy, and when other people project perceptions of AI onto users (Dang and Liu, 2025). In organizational systems, algorithms can treat workers as data points rather than as whole persons (Schultz et al., 2025), yet AI is sometimes given humanlike features, which can make humans seem less uniquely human relative to AI (Kim and McGill, 2025). These studies show that technology can address risks to organizational resilience while also creating new risks to humanness. Thus, a measure of humanization is needed to assess whether people perceive their humanness as affirmed within organizations particularly during disruptions and technological changes.

Organizational cultures have been described as humanizing people when they emphasize uniqueness, freedom, dignity, and sociability (Melé, 2003). When the organization is understood as a community of people, work becomes relational rather than purely economic (Melé, 2012). Humanistic management is an approach to management that treats people as whole, unique, free, and worthy of dignity (Ferris, 2013; Melé, 2016). This view rejects the reduction of people to fragmented preferences or functions in favor of an emphasis on the whole person (Domingo and Melé, 2022). Organizations that implement these types of human-centered approaches are referred to as human-centered organizations (HCOs), where dignity and related human factors are treated as central features of the organizational climate rather than peripheral concerns (Townsend and Romme, 2024).

Dehumanization involves denials of agency or autonomy, uniqueness or individuality, and moral worth (Haslam, 2006; Haslam and Loughnan, 2014; Kteily and Landry, 2022), which are the same dimensions found in research on humanness. The American Psychological Association's Dictionary of Psychology aligns with this by defining dehumanization as the denial of autonomy, individuality, and dignity specifically (American Psychological Association, 2018). Across these sources on humanness and its denial, autonomy, individuality, and dignity emerge as central to humanness and thus serve as a foundation of organizational humanization. These dimensions are not newly discovered constructs. Instead, they are grounded in established lines of research and measurement that have not been integrated into a higher-order framework. Thus, the proposed framework centers on meaningful influence, recognition of uniqueness, and recognition of worth. These dimensions define the scope of humanization as it is examined here rather than encompassing every favorable organizational experience. While autonomy, individuality, and dignity emerged as central to humanness and serve as a foundation, they should not be interpreted as representing the totality of humanness or humanization.

Autonomy is considered a basic psychological need in self-determination theory (Gagné and Deci, 2005; Ryan and Deci, 2000). This is consistent with humanistic management's treatment of people as free beings rather than passive resources (Melé, 2016). In work contexts, autonomy has been defined and measured across method, scheduling, and criteria: freedom in how work is done, when it is done, and how it is evaluated (Breaugh, 1985, 1999). Since organizational roles necessarily involve constraints, including a minimum expectation of the role being performed, autonomy in organizational humanization does not require absolute freedom. Instead, it reflects whether the person perceives meaningful influence over their activities within the organization.

Individuality means the uniqueness of a person. Optimal distinctiveness theory identifies competing needs for both uniqueness, or distinctiveness, and belonging (Brewer, 1991), suggesting that even within a group the individual needs to retain their individuality (Wojtyła, 1979). This is closely related to diversity as a range of individual differences, inclusion as whether those unique differences are valued and embraced through belongingness (Chung et al., 2020; Shore et al., 2011), and authenticity as whether a person can express their individuality (Jansen et al., 2014). Thus, individuality is a dimension of humanness, while inclusion, belongingness, and authenticity describe ways the affirmation of this dimension can be experienced in organizational contexts. However, belongingness alone does not establish that individuality is affirmed. For example, a person might be accepted as an insider while being expected to conform rather than express their uniqueness (Shore et al., 2011).

Dignity is the self- and other-recognized worth of a person in an organization, acquired or injured in work contexts through respectful interaction, recognition of competence and contribution, equality, and inherent value (Thomas and Lucas, 2019). Respect is therefore represented within dignity in the Organizational Humanization Scale (OHS) rather than included as a separate dimension alongside it. From a Kantian view, this worth is intrinsic, and organizations have a duty to protect and promote it (Pirson, 2017), which implies equal dignity among people. When people perceive violations of that equality at an organizational level, they also perceive lower dignity at the individual level (Sainz et al., 2023).

Integrating this body of research, I propose a definition of organizational humanization as the affirmation of autonomy, individuality, and dignity within an organization. This definition integrates research on humanness, dehumanization, humanistic management, and human-centered organizations. It gives the construct specificity for empirical examination and practical application while preserving its grounding in a holistic view of the person. It also provides a foundation for examining organizational humanization relative to other organizational constructs.

There are other constructs closely related to but distinct from perceived organizational humanization. Psychological safety is one such construct related to this framework, but it addresses a different question concerning whether people feel they are able to express themselves in their work roles without fearing harm to their self-image, status, or career (Kahn, 1990). This overlaps with the expression of individuality, but perceived safety alone does not establish that uniqueness is valued, meaningful influence is allowed, or worth is recognized. Psychological safety is thus a related construct rather than an additional dimension of organizational humanization.

Social capital and engagement are expected correlates of organizational humanization. At the social level, social capital refers to networks, norms, and trust that facilitate coordination and cooperation in a reciprocal way (Putnam, 1995). In organizations, it includes bonding, meaning within groups, and bridging, meaning between groups (Adler and Kwon, 2002). Research has identified social capital as an antecedent to organizational resilience during the pandemic through such relationships and dynamic capabilities (Ozanne et al., 2022), while engagement declined due to job insecurity and other factors (Hur, 2022; Ployhart et al., 2021). Engagement refers to the active investment of physical, cognitive, and emotional energy in work (Kahn, 1990), and it is commonly measured through vigor, dedication, and absorption (Schaufeli et al., 2002). Associations between these constructs and the dimensions of organizational humanization provide the foundation for a nomological network of organizational humanization.

Autonomy has established associations with both correlates and with the other dimensions of organizational humanization. The association between job autonomy and social capital exists in both bonding and bridging forms (Tsounis et al., 2023). At the team level, autonomy is linked with greater trust and network density, suggesting that shared freedom can strengthen relationships and attitudes (Yamaguchi, 2013). Autonomy has also shown direct and indirect effects on work engagement, partly through psychological meaningfulness (Sung et al., 2022). In essence, findings suggest that greater autonomy is associated with positive attitudes and stronger workplace relationships. Autonomy is also tied to dignity, as schedule instability is associated with lower dignity through reduced respect and recognition (Woods, 2026). Greater autonomy at work has also been associated with authenticity since autonomy allows people to act in ways consistent with their own ideas, goals, and talents (Maunz and Glaser, 2023).

Individuality is related to uniqueness, authenticity, inclusion, and belongingness. Work group inclusion is similarly defined through uniqueness and belongingness (Chung et al., 2020; Shore et al., 2011), which is conceptually similar to diversity and inclusion. Research has associated organizational practices emphasizing diversity and inclusion with climates of trust, social capital, and engagement (Downey et al., 2015; Moon, 2018), while similarly associating authenticity with engagement through self-determined motivation (Van den Bosch and Taris, 2018). Essentially, findings suggest that perceived expression of one's authentic self is associated with trust and engagement. Individuality is also associated with dignity, as perceived respect has been linked with feelings of inclusion and value in teams (Ellemers et al., 2013).

Dignity has been associated with higher engagement and lower burnout and turnover intentions (Thomas and Lucas, 2019), which suggests that perceived dignity is connected to whether people remain invested in their organization or withdraw from it. Similarly, during the pandemic, workplace dignity predicted later relatedness and social contribution, and relatedness predicted later dignity (Allan and Blustein, 2022). This suggests a reciprocal relationship between dignity and social factors that may reinforce one another over time.

The purpose of this study was to define organizational humanization and evaluate the Organizational Humanization Scale (OHS) as a brief measure of that construct. This study defines organizational humanization as the affirmation of autonomy, individuality, and dignity within an organization and operationalizes it as the perceived affirmation of those dimensions. The OHS therefore assesses individual perceptions rather than independently observed organizational practices. I conceptualized the OHS as a balance to the dehumanization literature and grounded it in established work on autonomy (Breaugh, 1985), work group inclusion (Chung et al., 2020), and workplace dignity (Thomas and Lucas, 2019). Accordingly, I designed this study as an integration and validation effort rather than as the development of an entirely separate theoretical domain.

This study had four aims. The first aim was to determine whether organizational humanization is best represented as a higher-order factor reflected by autonomy, individuality, and dignity, and whether that structure supports interpretation of both an overall humanization total score and individual subscale scores. The second aim was to evaluate the psychometric properties of the OHS, including internal consistency reliability, convergent validity, and internal discriminant validity. The third aim was to begin establishing a nomological network for organizational humanization by examining the OHS's associations with workplace social capital and work engagement. The fourth aim was to evaluate whether the OHS demonstrates patterns of association comparable to the established measures despite having meaningfully fewer items.

This study does not aim to define the totality of humanness or humanization. Its purpose is narrow and practical: to define and measure organizational humanization and provide evidence of its associations with organizational constructs identified in the context of the COVID-19 pandemic and the emergence of AI. This study also does not aim to replace the established measures it integrates, but to evaluate whether autonomy, individuality, and dignity function as related dimensions of a broader organizational humanization construct.

## Materials and methods

### Participants and procedure

In this validation study of an integrated measure of organizational humanization, I employed a cross-sectional survey design. The study involving human participants was reviewed and approved by the Liberty University Institutional Review Board (No. IRB-FY25-26-945). Participants provided written informed consent electronically before beginning the survey. No directly identifying information was collected from participants, and survey data were stored on a password-protected laptop. I recruited participants through the Prolific crowdsourcing platform because it allowed recruitment of a sufficiently large and demographically broad sample of full-time employed adults in the United States. I administered the survey through the Qualtrics survey platform. Eligible participants were full-time employed adults in the United States with at least 1 year in their current organization. Because the OHS concerns perceptions within an organization, this criterion focused the sample on employees with sustained exposure to its practices and interactions. The survey included 57 items across six measures, five demographic questions, and six attention checks, for a total of 68 questions. I randomized both the order of the measures and the order of items within measures. I placed an attention check between each measure.

Participants were compensated $3 based on an estimated completion time of 15 min. I targeted a sample size of 500 based on recommendations for structural equation modeling and confirmatory factor analysis (Bandalos, 2018; Muthén and Muthén, 2002; Wolf et al., 2013). Demographic variables included age, sex, industry, job level, and organization size. Job level options were junior, senior non-manager, manager, and executive. Organization size options were small (fewer than 20 employees), medium (20 to 1,000 employees), and large (more than 1,000 employees). Industry options were a broad range of sectors and an option for “other.”

### Measures

The Organizational Humanization Scale (OHS) is a 9-item measure of perceived affirmation of autonomy, individuality, and dignity in organizational contexts. Each dimension contains three items. The Autonomy items represent method, scheduling, and criteria autonomy (Breaugh, 1985). The Individuality items represent valuing uniqueness, embracing uniqueness, and allowed expression of uniqueness, based primarily on the Work Group Inclusion Measure (Chung et al., 2020). The Dignity items represent respectful interaction, respect for one's contribution, and equal respect, based on the respectful interaction, competence-contribution, and equality factors of the Workplace Dignity Scale (Thomas and Lucas, 2019). I theorized Humanization as a higher-order factor reflected by Autonomy, Individuality, and Dignity.

I adapted items from these established measures to preserve continuity with their respective content while integrating them under organizational humanization. The adaptation balanced coverage of the intended content with conciseness. I then revised wording to broaden the context from work specifically to organizational activities more generally. Specifically, I replaced “work” with “activities” to allow broader organizational application in non-work contexts. Finally, I used factor-specific stems to anchor items within their intended dimensions. For Autonomy items, the stem is “I am allowed to influence” because autonomy in organizational contexts is generally a matter of meaningful influence rather than absolute choice. For Individuality items, the stem is “My uniqueness” because individuality centers the person's distinctiveness. For Dignity items, the stem is “People show respect” because respect reflects active recognition of worth. I did not conduct a formal independent expert-rating procedure for the adapted OHS items. The complete OHS items and response options are provided separately in Supplementary Table 1.

To evaluate content preservation and comparative associations, I included the established measures on which I based the OHS items. These included the Work Autonomy Scales (WAS; Breaugh, 1985), the Work Group Inclusion Measure (WGIM; Chung et al., 2020), and the Workplace Dignity Scale (WDS; Thomas and Lucas, 2019). I reverse-scored the four WDS indignity items so higher scores reflected higher dignity. These were the only reverse-scored items. For analyses labeled as WDS Partial, I used the respectful interaction, competence-contribution, and equality factors of the full WDS because these corresponded with the three dignity items in the OHS. I included the Workplace Social Capital scale (WSC; Kouvonen et al., 2006) and the 3-item version of the Utrecht Work Engagement Scale (UWES-3; Schaufeli et al., 2019) as correlates for nomological validity testing.

### Analytic strategy

I analyzed the data in R version 4.5.1 released in 2025 using the tidyverse, careless, psych, lavaan, semTools, and BifactorIndicesCalculator packages. I screened responses for straightlining and failed attention checks prior to analysis, excluding any that failed either. I defined straightlining as selecting the same response across all items within a scale. This was assessed prior to reverse-scoring the WDS indignity items. I excluded responses if this occurred across three or more scales between the OHS, WAS, WGIM, WDS, and WSC, excluding the UWES-3 since it only included three items. To assess sensitivity to the straightlining screening, I repeated the confirmatory factor analysis, reliability, validity, structural equation modeling, and invariance analyses without the straightlining exclusion. Attention-check exclusions, item scoring, and model specifications were unchanged.

I used confirmatory factor analysis (CFA) with robust maximum likelihood estimation to evaluate the factor structure of the OHS. I assessed model fit using comparative fit index (CFI), Tucker-Lewis index (TLI), root mean square error of approximation (RMSEA), standardized root mean square residual (SRMR), Akaike information criterion (AIC), and Bayesian information criterion (BIC). I followed standard guidelines for factor analysis (Bandalos, 2018; Brown, 2015; Hu and Bentler, 1999; Kline, 2023) and evaluated models based on model fit, factor loadings, and theoretical interpretability. To evaluate the latent structure of the OHS, I tested four confirmatory factor analysis models. Model 1 (M1) was a unidimensional model in which all nine items loaded on a single Humanization factor. Model 2 (M2) was a correlated three-factor model with first-order factors of Autonomy, Individuality, and Dignity. Model 3 (M3) was a second-order model in which those three first-order factors loaded on a single second-order Humanization factor. Model 4 (M4) was a bifactor model with a general Humanization factor and three orthogonal specific factors of Autonomy, Individuality, and Dignity.

While the second-order model represented the theorized structure, I included the correlated three-factor model to compare the OHS factors with the three established measures in the same modeling form. Because a second-order model with exactly three first-order factors is equivalent in fit to the correlated factors model, I evaluated the models based on model fit, factor loadings, bifactor indices for the general factor, and theoretical interpretability rather than on fit alone. To evaluate the strength of the Humanization general factor and justify reporting a total score, I calculated explained common variance (ECV) and omega hierarchical (ω*h*) from the bifactor model (Reise et al., 2013; Rodriguez et al., 2016). I assessed internal consistency reliability using composite reliability (CR), Cronbach's alpha, and McDonald's omega.

To assess convergent validity and evidence of content preservation, I used average variance extracted (AVE) and Pearson correlations comparing the OHS Autonomy with the WAS, OHS Individuality with the WGIM, and OHS Dignity with the WDS. To assess internal discriminant validity, I used the Heterotrait-Monotrait ratio (HTMT) among the OHS subscales to evaluate whether Autonomy, Individuality, and Dignity remained empirically distinguishable within the integrated measure. I assessed nomological validity using structural equation modeling (SEM) with robust maximum likelihood estimation to examine associations with workplace social capital (WSC) and work engagement (UWES-3).

SEM1 represented the primary theoretical model, with Humanization as a second-order factor associated with both outcomes. SEM2 represented the bifactor model to examine whether the Humanization general factor primarily accounted for the associations with the outcomes when modeled alongside the specific factors. SEM3 represented the OHS correlated-factors model, with Autonomy, Individuality, and Dignity associated with both outcomes. SEM4 represented the established-measures comparison model, with WAS, WGIM, and WDS associated with both outcomes. SEM3 and SEM4 were intended as comparison models because both represented models with correlated factors associated with the same outcomes.

To assess sensitivity to potential common-method variance (CMV), I fit a supplementary unmeasured latent method-factor model (Podsakoff et al., 2003). This model, CMV1, retained SEM1 and added a factor with freely estimated loadings on all 20 OHS, WSC, and UWES-3 indicators. After examining CMV1, I conducted an analysis of a second model, CMV2, informed by the discussion of common response formats such as agreement and frequency (Podsakoff et al., 2024). The OHS and WSC used five-point agreement responses whereas UWES-3 used seven-point frequency responses. Common formats may encourage more consistent responding across items, although evidence concerning such effects is mixed. I therefore examined whether the findings depended on allowing the added influence to extend across these different response formats.

CMV2 restricted the added factor to the 17 agreement format indicators by fixing the three UWES-3 added factor loadings to zero. All constructs and observed indicators remained in both models. In both models, the added factor's variance was fixed to one, and it was specified as uncorrelated with Humanization and the residuals of Autonomy, Individuality, Dignity, Social Capital, and Engagement. The original structural paths and outcome residual covariance were retained. Using robust maximum likelihood estimation, I compared model fit, standardized structural paths and their 95% confidence intervals (CIs), and loadings, with and without each added factor in the primary analysis (*N* = 441) and the screening sensitivity analysis without straightlining exclusions (*N* = 494). I checked convergence and admissibility using lavaan's built-in functions.

Finally, I assessed two-group measurement invariance using multi-group CFA of the second-order model across age, sex, job level, and organization size at the configural, metric, and scalar levels (Bandalos, 2018). These analyses evaluated measurement comparability across the selected groups concerning item loadings and item intercepts, but I did not test substantive differences in group means or equality of second-order loadings. I also did not assess invariance across industries because group sizes were too small with no defensible way to combine them into appropriately sized groups. To maximize group sizes, I grouped age as 40 and above or under 40, job level as non-management or management, and organization size as 1,000 or fewer employees or over 1,000 employees. The sex-invariance analyses included male and female participants. The three participants who selected “Prefer not to say” were only excluded from the sex-invariance analyses. I interpreted changes in CFI, RMSEA, and SRMR less than 0.01 as support for invariance (Kline, 2023).

## Results

I collected survey responses from 501 full-time employed adults with at least 1 year of tenure in their current organization. All participants responded to all items and thus there were no missing data. I excluded 54 responses for straightlining across three or more scales, leaving 447 responses, and then excluded six additional responses for failing one of the attention checks. The primary analysis included 441 participants. Seven participants failed attention checks, but one was also excluded under the straightlining screen. The screening sensitivity analysis without straightlining exclusions included 494 participants. Table 1 summarizes participant demographics. The sample reflected a broad range of ages, job levels, organization sizes, and industries.

**Table 1:** Sample demographics.

Demographic	*n*	Percent
Age		
18–24	5	1.1
25–34	88	20.0
35–44	180	40.8
45–54	111	25.2
55–64	46	10.4
65+	11	2.5
Sex		
Male	255	57.8
Female	183	41.5
Prefer not to say	3	0.7
Job level		
Junior	120	27.2
Senior (non-manager)	108	24.5
Manager	189	42.9
Executive	24	5.4
Industry		
Information technology	65	14.7
Educational services	51	11.6
Healthcare and social assistance	50	11.3
Retail or wholesale trade	44	10.0
Finance and insurance	37	8.4
Manufacturing	33	7.5
Other	161	36.5
Organization size		
Between 20 and 1,000 employees	215	48.8
More than 1,000 employees	170	38.5
Less than 20 employees	56	12.7

Table 2 presents descriptive statistics for the study scales. Scale means were above their midpoints and standard deviations were moderate. Skewness and kurtosis were also low to moderate and below absolute values that are sometimes used to indicate severe univariate non-normality (Kline, 2023). Table 3 presents the model fit indices, including CFI, TLI, RMSEA, SRMR, AIC, and BIC. As expected, M2 and M3 were identical in fit because a second-order model with three first-order factors is equivalent in fit to the corresponding correlated first-order factors model. M1 fit poorly, whereas M2, M3, and M4 each fit well and comparably.

**Table 2:** Descriptive statistics for scale totals.

Scale	*n*	Mean	SD	Skew	Kurt
Organizational humanization scale (OHS)	441	3.82	0.76	−1.15	1.88
Work autonomy scales (WAS)	441	4.90	1.34	−0.99	0.57
Work group inclusion measure (WGIM)	441	3.87	0.72	−1.17	2.06
Workplace dignity scale (WDS)	441	5.69	1.08	−1.45	2.23
Workplace social capital scale (WSC)	441	3.97	0.76	−1.26	1.99
Utrecht work engagement scale (UWES)	441	4.57	1.29	−0.56	0.24

SD, standard deviation; Skew, skewness; Kurt, kurtosis.

**Table 3:** CFA model fit indices.

Model	χ^2^	*df*	*p*	CFI	TLI	RMSEA	SRMR	AIC	BIC
M1	381.22	27	< 0.001	0.82	0.75	0.22	0.09	8,463	8,537
M2	47.60	24	0.003	0.99	0.98	0.05	0.03	7,935	8,021
M3	47.60	24	0.003	0.99	0.98	0.05	0.03	7,935	8,021
M4	31.49	18	0.025	0.99	0.99	0.05	0.02	7,926	8,036

χ^2^, scaled chi-square statistic; df, degrees of freedom; CFI, comparative fit index; TLI, Tucker-Lewis index; RMSEA, root mean square error of approximation; SRMR, standardized root mean square residual; AIC, Akaike information criterion; BIC, Bayesian information criterion.

Table 4 shows standardized factor loadings. All item loadings were strong and ranged from 0.79 to 0.93. The second-order factor loadings were also strong for Autonomy (0.83), Individuality (0.93), and Dignity (0.80). From M4, ECV was 0.73 and ω_*h*_ was 0.85. Since M2 and M3 were identical in fit, I retained M3 as the primary representation of the OHS on theoretical grounds rather than superior model fit. The strong second-order loadings and bifactor indices supported interpretation of a Humanization total score. I retained M2 and M4 for later SEM comparisons.

**Table 4:** Standardized factor loadings for OHS items in the second-order model.

Item	Autonomy	Individuality	Dignity
I am allowed to influence…			
how I do my activities here.	0.84		
when I do my activities here.	0.84		
how my activities are evaluated here.	0.79		
My uniqueness…			
is valued here.		0.93	
is embraced here.		0.91	
can be expressed here.		0.85	
People show respect…			
in interactions with me here.			0.87
for what I do here.			0.87
to me the same as they do to others here.			0.83

Table 5 shows reliability estimates for all scales. The OHS and WGIM are shown by subscale and total scores. WDS Partial represents respectful interaction, competence-contribution, and equality factors of the full scale, which correspond with the dignity items in the OHS. All values were strong and supported strong reliability for the OHS and its subscales.

**Table 5:** Reliability estimates for study scales.

Scale	*k*	Alpha	Omega	CR
OHS autonomy	3	0.86	0.86	0.86
OHS individuality	3	0.93	0.93	0.93
OHS dignity	3	0.89	0.89	0.89
OHS total	9	0.93	0.93	0.85
WAS	9	0.94	0.94	
WGIM uniqueness	5	0.87	0.87	
WGIM belongingness	5	0.91	0.91	
WGIM total	10	0.93	0.93	
WDS partial	8	0.95	0.95	
WDS total	18	0.97	0.97	
WSC	8	0.93	0.93	
UWES	3	0.89	0.89	

*k*, number of items; CR, composite reliability.

AVE values were strong for Autonomy (0.67), Individuality (0.81), and Dignity (0.73), all of which were above the generally accepted 0.50 cutoff. As shown in Table 6, all scales were correlated with each other and statistically significant at *p* < 0.001. The OHS subscales correlated strongest with their respective established measures. OHS Autonomy correlated strongest with the WAS (*r* = 0.79) and OHS Dignity correlated strongest with the WDS (*r* = 0.85). The WGIM correlated equally with OHS Individuality (*r* = 0.76) and OHS Dignity (*r* = 0.76), indicating that convergence with the intended dimension was strong but not exclusive. Overall, these results support convergent validity and suggest that the integrated measure retained continuity with the content of the established measures from which its dimensions were adapted.

**Table 6:** Intercorrelations among study scales.

Scale	1	2	3	4	5	6	7	8	9	10	11	12
1. OHS	–											
2. Autonomy	0.88	–										
3. Individuality	0.91	0.70	–									
4. Dignity	0.84	0.58	0.67	–								
5. WAS	0.70	0.79	0.57	0.44	–							
6. Uniqueness	0.73	0.57	0.67	0.69	0.46	–						
7. Belongingness	0.77	0.57	0.74	0.73	0.45	0.76	–					
8. WGIM	0.80	0.61	0.76	0.76	0.49	0.93	0.95	–				
9. WDS partial	0.82	0.60	0.73	0.86	0.49	0.70	0.81	0.81	–			
10. WDS	0.81	0.58	0.74	0.85	0.47	0.68	0.81	0.80	0.97	–		
11. WSC	0.74	0.54	0.68	0.75	0.41	0.69	0.80	0.80	0.82	0.82	–	
12. UWES	0.58	0.47	0.57	0.49	0.41	0.49	0.62	0.60	0.58	0.56	0.52	–

All correlations were statistically significant at *p* < 0.001.

HTMT values among the three OHS dimensions were all below the 0.85 cutoff (Henseler et al., 2015) for Autonomy-Individuality (0.78), Autonomy-Dignity (0.66), and Individuality-Dignity (0.74). These results support internal discriminant evidence among Autonomy, Individuality, and Dignity, indicating that the dimensions were distinct enough to support separate subscale interpretation while still cohering under organizational humanization.

I assessed nomological validity using four SEMs examining associations with workplace social capital and work engagement. SEM1 tested whether the higher-order Humanization factor was associated with the outcomes, SEM2 tested whether the Humanization general factor accounted for these associations when modeled alongside the specific factors, and SEM3 and SEM4 compared the OHS correlated-factors model with the established-measures model. Figures 1–4 show standardized path coefficients with solid lines indicating statistical significance (*p* < 0.05) and dashed lines indicating non-significance (*p* ≥ 0.05).

**Figure 1:**
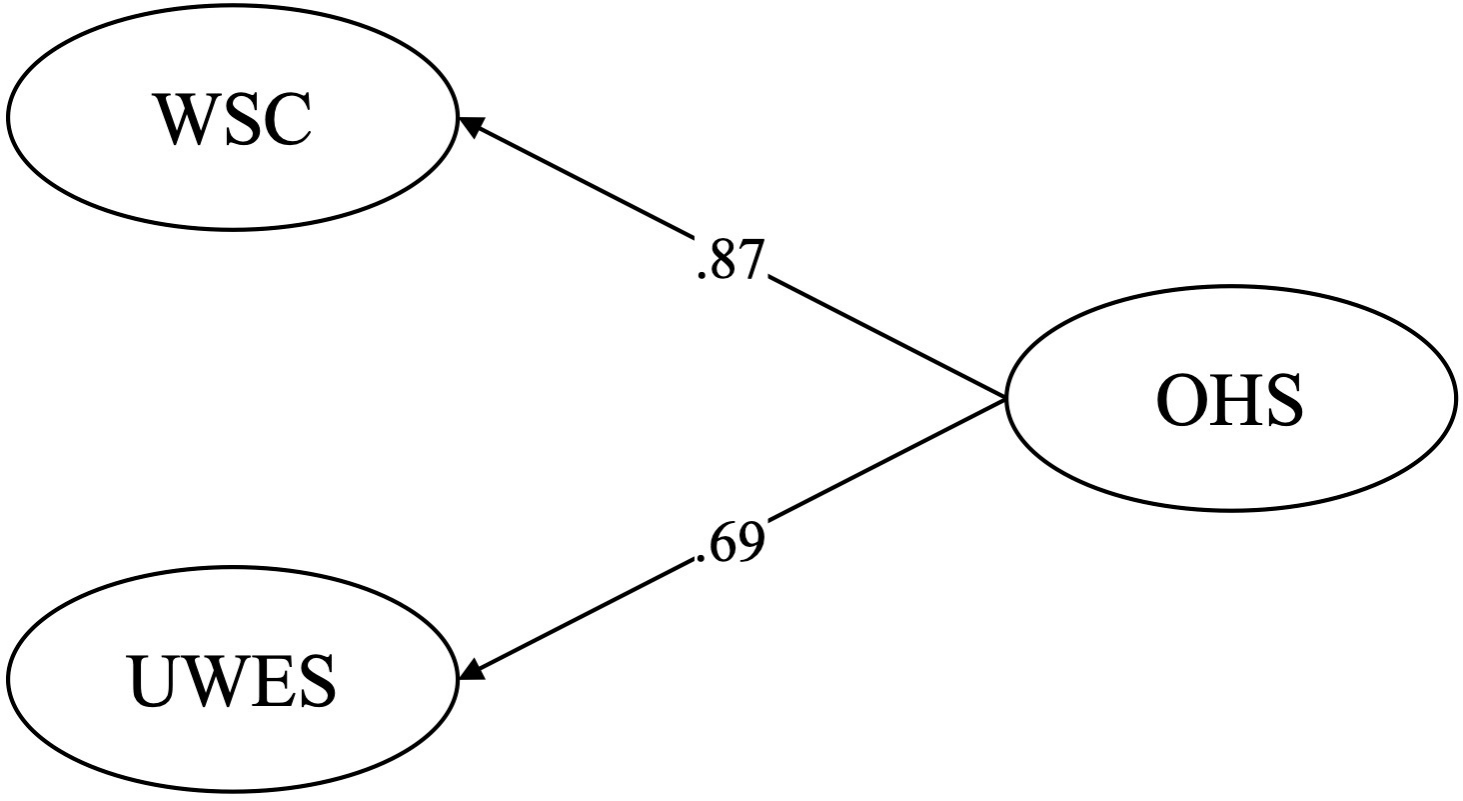
Structural equation model of humanization and outcomes.

**Figure 2:**
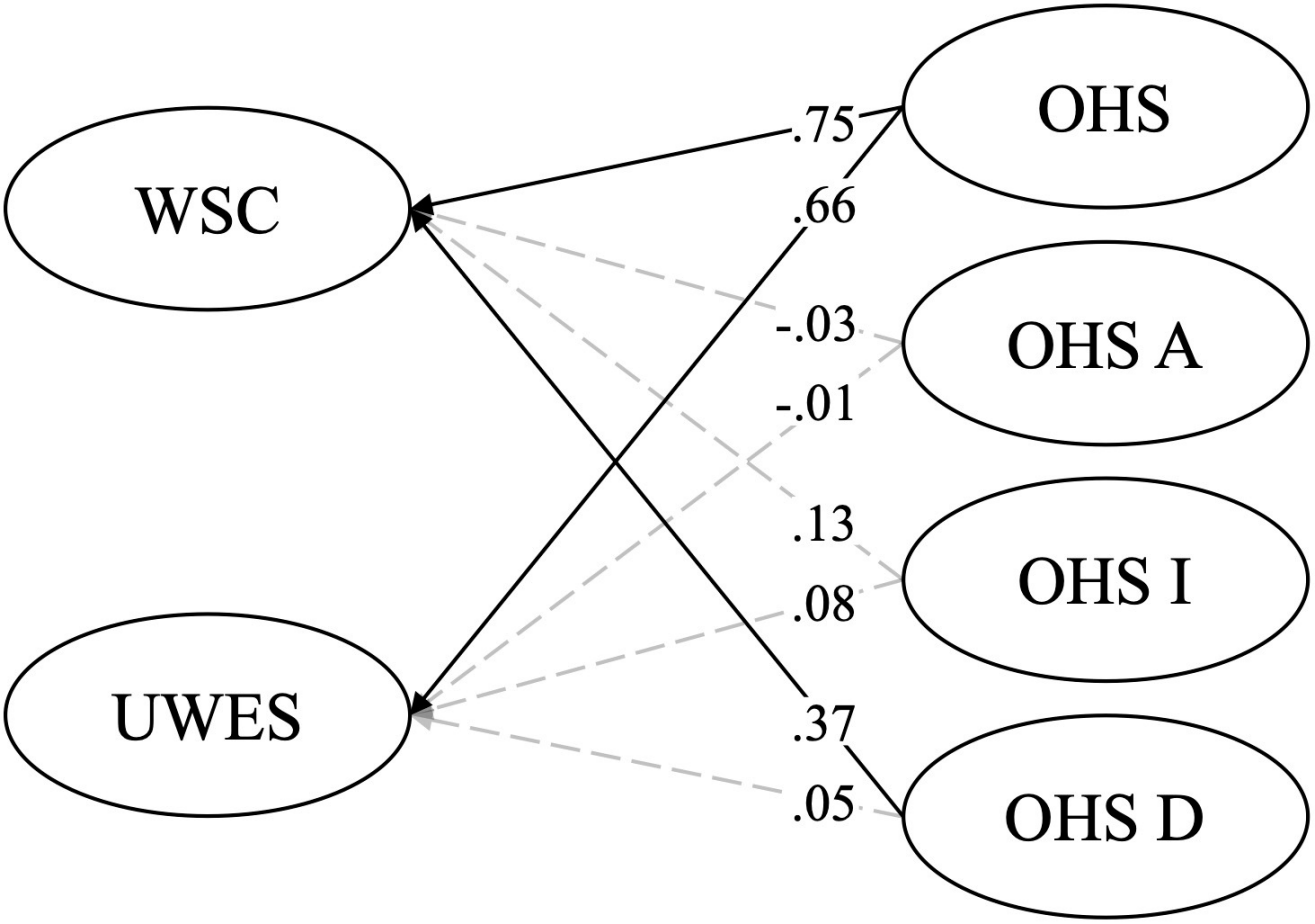
Structural equation model of the general factor, specific factors, and outcomes.

**Figure 3:**
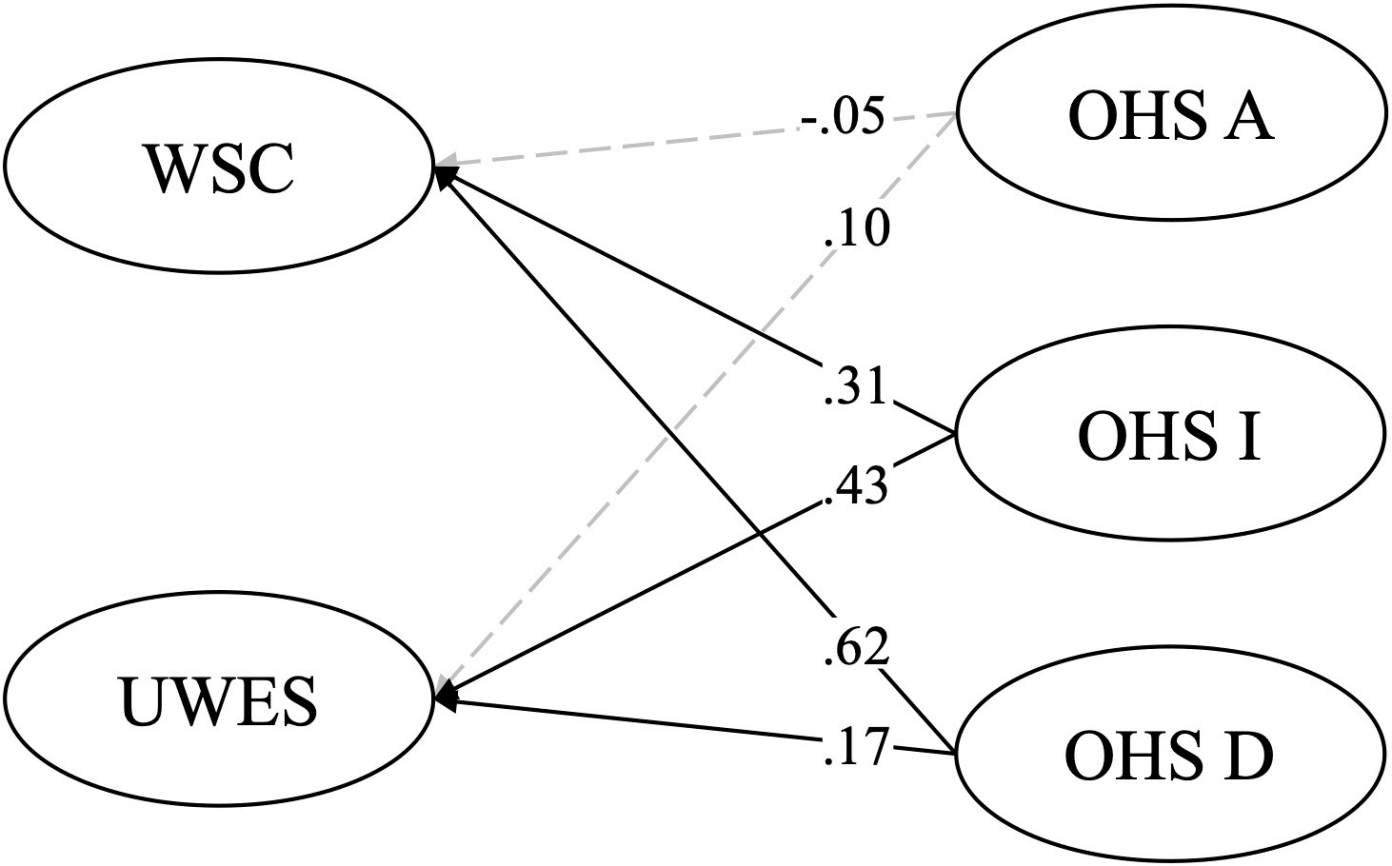
Structural equation model of correlated factors and outcomes.

**Figure 4:**
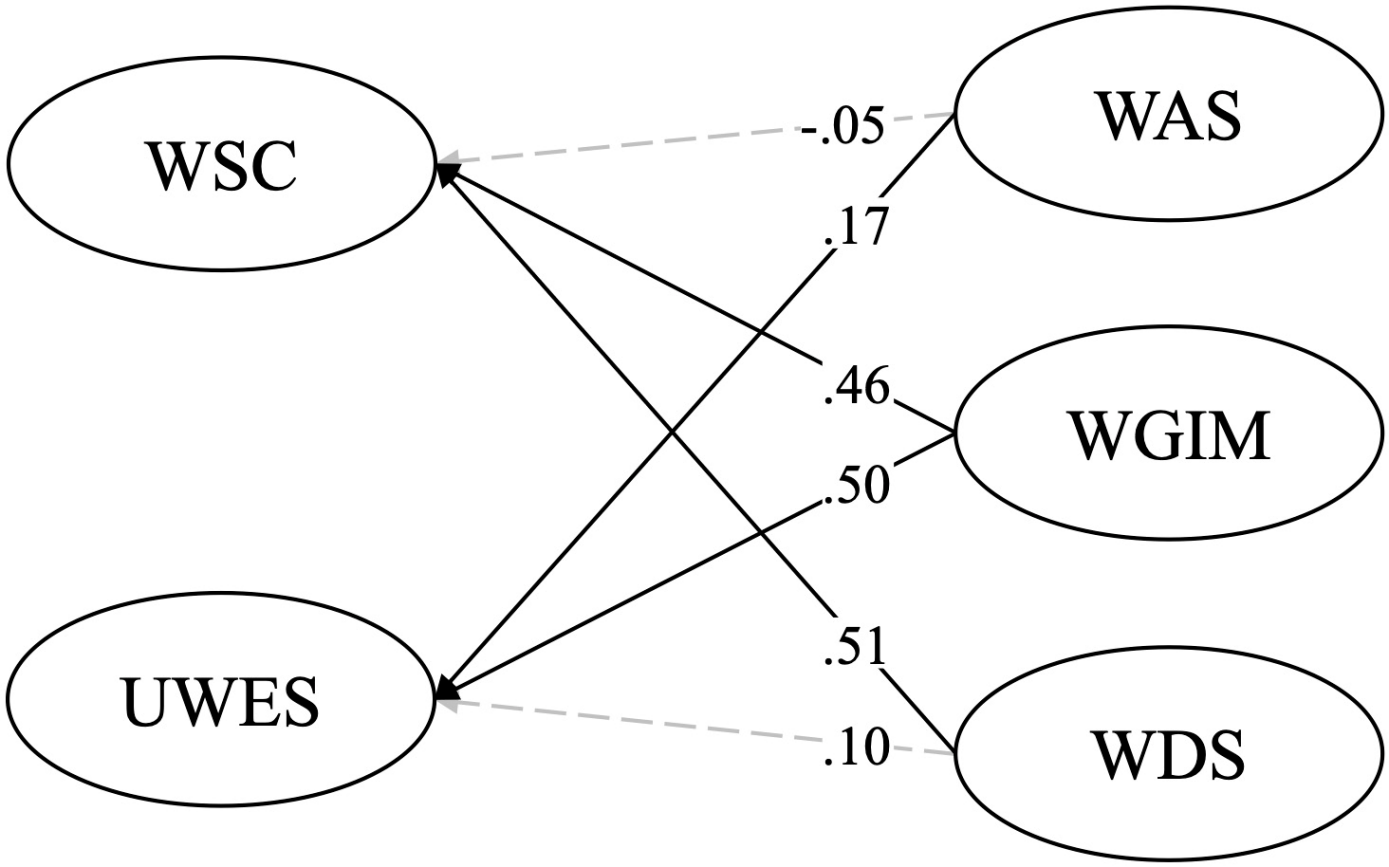
Structural equation model of established measures and outcomes.

In SEM1, Humanization was strongly associated with social capital (β = 0.87, *p* < 0.001) and engagement (β = 0.69, *p* < 0.001), accounting for 75% of the variance in social capital (*R*^2^ = 0.75) and 47% of the variance in engagement (*R*^2^ = 0.47). In SEM2, Humanization was strongly associated with social capital (β = 0.75, *p* < 0.001) along with Dignity (β = 0.37, *p* < 0.001), but the other specific factors were nonsignificant. Humanization was also strongly associated with engagement (β = 0.66, *p* < 0.001), but the three specific factors were again non-significant. The predictors in SEM2 accounted for 72% of the variance in social capital (*R*^2^ = 0.72) and 44% of the variance in engagement (*R*^2^ = 0.44). Together, SEM1 and SEM2 indicate that the shared variance among Autonomy, Individuality, and Dignity was substantially associated with both outcomes through the Humanization general factor.

In SEM3, Individuality was the strongest factor associated with engagement (β = 0.43, *p* < 0.001), followed by Dignity (β = 0.17, *p* = 0.026), while Autonomy was non-significant. For social capital, Dignity was the strongest factor (β = 0.62, *p* < 0.001), followed by Individuality (β = 0.31, *p* < 0.001), while Autonomy was again non-significant. Together, Autonomy, Individuality, and Dignity accounted for 72% of the variance in social capital (*R*^2^ = 0.72) and 42% of the variance in engagement (*R*^2^ = 0.42). In SEM4, WGIM (β = 0.46, *p* < 0.001) and WDS (β = 0.51, *p* < 0.001) were associated with social capital, while WAS was non-significant. For engagement, WGIM (β = 0.50, *p* < 0.001) and WAS (β = 0.17, *p* = 0.003) were both associated with engagement, while WDS was non-significant. Together, WAS, WGIM, and WDS accounted for 83% of the variance in social capital (*R*^2^ = 0.83) and 47% of the variance in engagement (*R*^2^ = 0.47).

Table 7 shows model fit indices for these four SEM models. SEM1, SEM2, and SEM3 all fit well. SEM4 had worse fit with poor CFI and TLI, but acceptable RMSEA and SRMR, and was thus interpreted cautiously. Together with the strong associations between Humanization and both outcomes, these results provide initial nomological evidence for the OHS. SEM1 and SEM2 indicated that the Humanization general factor accounted for the primary associations with workplace social capital and engagement, while SEM3 showed the OHS produced a similar pattern of associations relative to the established measures in SEM4.

**Table 7:** SEM model fit indices.

Model	χ^2^	*df*	*p*	CFI	TLI	RMSEA	SRMR	AIC	BIC
SEM 1	446.47	164	< 0.001	0.95	0.94	0.07	0.05	18,642	18,830
SEM 2	392.76	152	< 0.001	0.95	0.94	0.07	0.04	18,589	18,827
SEM 3	407.94	160	< 0.001	0.95	0.94	0.07	0.04	18,595	18,799
SEM 4	2,923.34	1,070	< 0.001	0.88	0.87	0.07	0.05	50,787	51,221

χ^2^, scaled chi-square statistic; df, degrees of freedom; CFI, comparative fit index; TLI, Tucker-Lewis index; RMSEA, root mean square error of approximation; SRMR, standardized root mean square residual; AIC, Akaike information criterion; BIC, Bayesian information criterion.

Table 8 reports group sizes for the measurement invariance analyses. Table 9 shows the measurement invariance results. Across age, sex, job level, and organization size, changes in CFI, RMSEA, and SRMR were all less than 0.01, supporting item-level measurement invariance across these groupings.

**Table 8:** Group sizes for measurement invariance.

Level	*n*
Age	
40 and above	248
Under 40	193
Sex	
Female	183
Male	255
Job level	
Non-management	228
Management	213
Organization size	
1,000 or fewer employees	271
Over 1,000 employees	170

**Table 9:** Measurement invariance across age, sex, job level, and organization size.

Model	CFI	RMSEA	SRMR	ΔCFI	ΔRMSEA	ΔSRMR
Age						
Configural	0.99	0.06	0.03			
Metric	0.99	0.05	0.03	0.002	−0.009	0.002
Scalar	0.99	0.05	0.03	0.000	−0.001	0.001
Sex						
Configural	0.99	0.07	0.03			
Metric	0.98	0.06	0.04	−0.002	−0.001	0.007
Scalar	0.98	0.06	0.04	−0.001	−0.002	0.001
Job level						
Configural	0.99	0.06	0.03			
Metric	0.99	0.06	0.04	0.001	−0.006	0.007
Scalar	0.99	0.05	0.04	0.000	−0.002	0.001
Org size						
Configural	0.99	0.06	0.03			
Metric	0.99	0.06	0.04	−0.001	−0.002	0.008
Scalar	0.99	0.06	0.04	−0.002	0.001	0.002

CFI, comparative fit index; RMSEA, root mean square error of approximation; SRMR, standardized root mean square residual. Δ indicates change from the preceding, less constrained model.

In the screening sensitivity analysis (*N* = 494), the measurement and nomological conclusions were maintained. The second-order model retained good fit (CFI = 0.99, TLI = 0.98, RMSEA = 0.06, SRMR = 0.02), item loadings ranged from 0.81 to 0.94, second-order loadings ranged from 0.82 to 0.94, AVE values remained above 0.50, and HTMT values remained below 0.85. In SEM1, Humanization remained positively associated with social capital (β = 0.88) and engagement (β = 0.72), both *p* < 0.001. The pattern of specific-factor associations in SEM2 and the measurement-invariance conclusions also remained unchanged.

In the primary analysis, both common factor models improved fit relative to SEM1. CMV1 provided the strongest global fit (CFI = 0.993, RMSEA = 0.028, SRMR = 0.024), followed by CMV2 (CFI = 0.985, RMSEA = 0.040, SRMR = 0.038), compared with SEM1 (CFI = 0.946, RMSEA = 0.072, SRMR = 0.047). In CMV1, the path between Humanization and social capital decreased from β = 0.866 to 0.041 (95% CI [−0.259, 0.341], *p* = 0.788) whereas the path between Humanization and engagement decreased from β = 0.685 to 0.447 but remained significant (95% CI [0.327, 0.567], *p* < 0.001). CMV2 retained both associations close to their primary analysis estimates, with β = 0.859 for social capital (95% CI [0.799, 0.920]) and β = 0.687 for engagement (95% CI [0.619, 0.755]), both *p* < 0.001.

The loading patterns also differed between specifications. In CMV1, three supervisor WSC items—WSC1, WSC2, and WSC8—retained positive loadings on Social Capital (0.328–0.462), while the work unit items—WSC3 through WSC7—had near-zero or negative loadings (−0.218 to −0.011). All eight WSC items loaded strongly and positively on the added factor (0.704–0.834). Dignity's second-order loading on Humanization decreased from 0.877 to 0.436. In contrast, CMV2 preserved the original measurement structure including positive WSC loadings ranging from 0.662 to 0.858 compared with 0.666 to 0.843 in SEM1. The added factor in CMV2 had small loadings on the OHS items (0.007–0.110) and opposing signs for the supervisor and work unit WSC items.

The screening sensitivity analysis (*N* = 494) showed the same pattern. The social capital path attenuated under CMV1 (β = 0.015, *p* = 0.924) but remained strong under CMV2 (β = 0.876, *p* < 0.001). Engagement remained positively associated with Humanization under both CMV1 (β = 0.451) and CMV2 (β = 0.717), both *p* < 0.001. The substantive and added factor loading patterns were also similar across the two screening conditions.

## Discussion

The purpose of this study was to define organizational humanization and evaluate the Organizational Humanization Scale (OHS) as a brief measure of that construct. I used a sample of 441 full-time employees in the U.S. across dozens of industries with at least 1 year of tenure. I proposed a definition of organizational humanization by integrating research on philosophical humanness, psychological dehumanization, and humanistic management, and I derived the OHS items from established measures of work autonomy, work group inclusion, and workplace dignity. The results supported the theorized higher-order structure, with autonomy, individuality, and dignity functioning as related but distinct dimensions of a broader organizational humanization construct.

The results provided strong psychometric evidence for the OHS. The OHS subscales correlated most strongly with the established measures corresponding to their intended content, suggesting that the scale retained continuity with the intended content of autonomy, individuality, and dignity. The higher-order structure was consistent with the theoretical interpretation that these dimensions cohered under a broader organizational humanization construct while the correlated first-order representation remained a fit-equivalent alternative. The first-order factors were strongly related, supporting interpretation of a Humanization total score while remaining distinct enough to support separate subscale interpretation. Together, the convergent, internal discriminant, second-order, and bifactor evidence supported the OHS as an integrated reflective measure.

As previously discussed, studies have linked autonomy, individuality, and dignity separately with social capital and engagement. These findings suggest that when these dimensions are integrated under organizational humanization, the expected associations remain. Perceived organizational humanization was strongly associated with workplace social capital and work engagement in the primary analysis, indicating that perceived humanization relates to both relational and motivational outcomes. In SEM2, when modeled alongside autonomy, individuality, and dignity, the humanization general factor accounted for most of the association with both outcomes. Only dignity retained a unique association with social capital beyond general humanization.

One possible explanation is that dignity might capture relational content that remains relevant after accounting for general humanization. The WSC includes supervisor consideration, concern for employee rights, mutual acceptance, and cooperation (Kouvonen et al., 2006). Dignity more directly concerns recognition of one's worth through respectful interaction, recognition of contribution, and equal treatment (Thomas and Lucas, 2019). These experiences might relate to the individual's perceptions of trust and cooperation in relationships within the group and with supervisors. The proximity of these constructs and possible overlap in item content might help explain dignity's retained association with social capital. It is also consistent with previous research showing that workplace dignity and relatedness can reinforce one another (Allan and Blustein, 2022). The non-significant specific-factor paths for autonomy and individuality do not mean those dimensions were unrelated to social capital since both had positive observed-score correlations with WSC.

The OHS therefore showed comparable nomological associations to the combined established measures while using substantially fewer items, suggesting that the adapted items retained continuity with the established measures without substantially narrowing or broadening their empirical associations. These findings provide an initial foundation for a nomological network of organizational humanization.

The large associations seen in these results, particularly between the OHS and WSC, might partly reflect that employees draw on the same workplace experiences when responding to these items. The OHS focuses on whether the person perceives their autonomy, individuality, and dignity as affirmed, while the WSC focuses on the perceived qualities of relationships within the work group and with supervisors. For example, an individual might perceive that coworkers respect their competence and contributions, reflecting an aspect of dignity. Those same interactions may also inform perceptions that people in the group take one another's contributions seriously and cooperate, reflecting aspects of social capital. Such interactions could produce closely corresponding responses about perceived affirmation and relationship quality even though the judgements address different content. Shared evaluative tendencies could also inflate these associations (Podsakoff et al., 2003, 2024), so their magnitude should also be interpreted alongside the common-factor sensitivity findings.

### Limitations and future directions

As with all research, this study has some limitations. First, the cross-sectional and self-report design limits causal interpretation. Organizational humanization was strongly associated with workplace social capital and work engagement, but the data cannot establish a causal direction or whether there could be a reciprocal relationship. The same-source design also leaves associations sensitive to common-method influence. A supplementary analysis showed that the association between the OHS and social capital was sensitive to how shared variance was represented. CMV1 provided stronger global fit but substantially changed the remaining Humanization and Social Capital loadings. The opposing supervisor and work unit loadings suggest a contrast between relationships consistent with the linking, bonding, and bridging content identified in the WSC's development (Kouvonen et al., 2006). Together with the reduced dignity loading, this complicates interpreting the attenuated path as a direct correction of the originally intended relationship. CMV1 does not by itself establish that humanization is unrelated to social capital.

CMV2 preserved the measurement structure and the associations between the OHS and both engagement and social capital. However, CMV2 assumes that the added common method influence does not directly affect engagement responses. Neither CMV1 nor CMV2 independently identifies the added factor as purely methodological since unmeasured factors can capture substantive and method-related covariance (Podsakoff et al., 2003, 2024). Overlapping relational content in OHS and WSC provides a plausible substantive contributor, but content and response-format differences cannot be separated here. The positive primary analysis associations therefore remain part of the initial validation evidence, while CMV1's stronger fit and substantial attenuation leave the role of shared method influences in the social capital relationship unresolved.

Second, the sample included full-time employed adults in the U.S. with a balanced sex distribution and a wide range of ages, industries, job levels, and organization sizes. Recruitment through Prolific involved self-selection into paid online research rather than probability sampling, so while the sample is demographically broad it does not establish the full breadth of representativeness. Prolific has shown strong data quality (Peer et al., 2022), but possible influences of self-selection were not directly assessed in this study. Invariance testing used broad two-group comparisons and did not assess industry invariance or equality of second-order loadings across groups. Measurement comparability across industries thus remains unestablished. Differences in work roles or organizational settings could affect interpretation, so cross-industry comparisons require additional evidence of measurement comparability. Thus, the findings of this study provide initial validation evidence rather than full evidence of generalizability across cultures, demographics, or non-work organizations.

Third, this study did not include a dehumanization measure, so it did not empirically test whether humanization and dehumanization are distinguishable constructs rather than opposite ends of a single continuum. While humanization and dehumanization are different theoretical constructs, they should nevertheless be empirically examined. Future studies should administer the OHS alongside established dehumanization measures to evaluate discriminant validity and directly compare these representations. Attention should be paid to the conceptual basis and item content of the dehumanization measure selected for comparison.

Fourth, the adapted OHS items were not evaluated through formal independent expert content ratings. The established source measures provided a foundation for item development, but do not by themselves establish the content validity of the adapted item set. Future studies should obtain expert ratings of item relevance, clarity, and coverage of autonomy, individuality, and dignity. Fifth, the criteria used in the nomological analyses were self-reported by the same participants. No supervisor ratings or organizational materials were included, so associations with criteria obtained from other sources remain untested. Sixth, the OHS was administered at one time point. Test-retest reliability was not assessed, so temporal stability of its scores remains unknown.

Future research should build on this initial validation evidence in several ways. First, researchers should strengthen evidence for the OHS and its generalizability by replicating the OHS factor structure, reliability, validity evidence, and invariance evidence across broader samples, including different countries, industries, job types, employment arrangements, and organizational sizes. This should include repeated OHS assessments, qualitative research on perceived humanization, and organizational records of policies and practices to examine whether adoption or enhancement of affirming practices is associated with increases in perceived affirmation. Multimethod studies should examine associations between the OHS dimensions and supervisor-related and work unit social capital across organizational contexts. Such studies should use different sources and response formats to distinguish relationships between constructs from shared item content and method influences.

Second, researchers should use longitudinal studies to examine directionality, temporal stability, and sensitivity to change. Such designs could examine whether humanization predicts later social capital, engagement, or other constructs, whether those outcomes shape later perceptions of humanization, or whether reciprocal relationships exist among them. Third, researchers should test the OHS in non-work organizations. Because the items refer to “activities” rather than “work,” the measure was designed for broader organizational use, but this requires validation in contexts not explored in this study.

### Theoretical and practical implications

The primary contribution of this study is an integrated definition of organizational humanization and the first measure of that construct. While humanness and human-centered organizations have been described conceptually, prior to this study there was no definition or measure of organizational humanization to balance the well-developed body of research on dehumanization. As a consequence, related research and practice remained fragmented across separate constructs. The OHS addresses this gap by integrating the fragmented research into a definition that aligns with the current body of knowledge and a measure that is brief and can be applied practically. This provides a foundation to examine organizational humanization through a concise but holistic lens, expanding knowledge on the positive balance to dehumanization.

For humanistic management and human-centered organizations, the OHS provides a measure to connect their emphasis on the whole person with evidence about employees' perceived affirmation (Melé, 2016; Townsend and Romme, 2024). Research could examine organizational policies and practices alongside the OHS to assess whether they are reflected in employee experiences across autonomy, individuality, and dignity. This can help distinguish an organization's intentions from employees' perceptions. However, it is important for practitioners to understand that the OHS concerns perceptions and thus it cannot serve on its own as an objective organizational auditing tool. In essence, it measures individual perceptions, not specifically what the organization is or is not doing to affect those perceptions.

The OHS could also be used alongside measures of other potentially related constructs to examine where content and associations overlap or differ. Psychological safety is one example. An employee might feel that they are able to express their ideas without fearing harm to their status or career (Kahn, 1990), but that does not inherently mean that they feel their ideas or uniqueness would be valued. Examining both the OHS and psychological safety could help distinguish perceived safety in self-expression from perceived affirmation of individuality, including whether expression of one's uniqueness is allowed. An individual might perceive that self-expression is allowed without feeling it is safe to do so due to anticipating negative consequences. Similar comparisons with other related constructs could clarify the boundaries of organizational humanization and whether the OHS provides information beyond established measures.

The integration of prior research supports organizational humanization as a broader higher-order construct reflected through autonomy, individuality, and dignity, while also giving broader context to research on autonomy, individuality, dignity, social capital, and engagement. The OHS was derived from established measures of work autonomy (Breaugh, 1985), work group inclusion (Chung et al., 2020), and workplace dignity (Thomas and Lucas, 2019), and was examined in relation to workplace social capital (Kouvonen et al., 2006) and work engagement (Schaufeli et al., 2019). Prior studies had linked these constructs in separate lines of work, but they had not been integrated under a higher-order organizational humanization construct and examined in relation to social capital and engagement. The present findings indicate that these dimensions not only remain distinguishable after integration but also share substantial variance through the broader Humanization factor. This study provides a broader context within which those prior associations can be examined.

By establishing the initial foundation for a nomological network of organizational humanization, this study provides initial evidence relevant to a question posed by Melé (2003) on whether humanizing organizations generate social capital. While this study cannot infer causality, the findings indicate that perceived organizational humanization is strongly associated with social capital in the primary analysis. Organizational resilience concerns how organizations maintain and adapt through disruptions and is distinct from individual resilience (Su and Junge, 2023). Social capital can provide access to knowledge and resources through relationships that support responses to disruption (Ozanne et al., 2022). The associations seen in this study suggest that perceived organizational humanization might be related to the relational aspects through which people exchange knowledge and coordinate responses to disruptions. This provides a theoretical link to examine whether perceived organizational humanization not only relates to social capital, but also to subsequent organizational resilience.

AI-driven digital transformation provides another context for the OHS and its associations with related constructs. AI could change how activities are assigned, executed, and evaluated, which directly relates to employees' influence and recognition of their contributions as humans (Schultz et al., 2025). The OHS provides a way to examine whether employees experience these changes as affecting their autonomy, individuality, and dignity. The positive association with engagement makes the OHS relevant for research into whether perceived affirmation during AI implementation accompanies sustained engagement with work. While these are relevant topics, neither AI implementation nor organizational resilience was measured here. Instead, the OHS and its associations with social capital and engagement set a foundation for future research in these directions.

Practically, the OHS gives organizations a brief way to assess perceived humanization without requiring a long survey that risks fatigue. However, its scores should not be equated with an objective audit of organizational policies and practices. The OHS contains only nine items and supports both a total score and separate autonomy, individuality, and dignity subscale scores. Since the scale uses a 1 to 5 response format, the score is both simple and interpretable while retaining a strong psychometric foundation. This is particularly useful for small or resource-limited organizations or groups within organizations that may not have the capacity to administer and maintain long measures or custom instruments.

The OHS also provides an assessment for understanding how people perceive humanization within their organization. Many organizational measures focus on outcomes or indicators such as engagement, trust, reciprocity, culture, climate, turnover intentions, burnout, satisfaction, meaningfulness, or performance. The OHS complements these measures by directly assessing perceived affirmation of autonomy, individuality, and dignity. The OHS can help organizations identify perceived issues to explore in relation to policies, practices, leadership, work designs, and change efforts. This may be especially useful before and during disruptions, restructuring, rapid growth, AI adoption, or similar contexts when organizational decisions may unintentionally reduce perceived humanization.

The total score and subscales provide different levels of practical interpretation. The total score indicates whether employees perceive the organization as humanizing overall, while the subscales help identify whether that perception is stronger or weaker for autonomy, individuality, or dignity. A dominant general factor does not mean that the total score should replace subscales completely (Rodriguez et al., 2016). Lower autonomy scores may point to concerns with influence over activities, timing, methods, or evaluation. Lower individuality scores may point to concerns with authenticity or inclusion-related experiences. Lower dignity scores may point to concerns with respect, recognition, or equal treatment. The dignity-specific association in SEM2 shows potential explanatory value beyond general humanization.

The OHS should not be treated as a complete intervention by itself, but as a starting point for inquiry and organizational learning. Together, the definition, measure, and initial validity evidence provide a foundation for studying and applying organizational humanization as a positive balance to dehumanization.

## Data Availability

The datasets presented in this study can be found in online repositories. The names of the repository/repositories and accession number(s) can be found below: Open Science Framework (OSF): https://osf.io/vtegy?view_only=ee1832c0e5fc4ccf815228a5e26d3a00.
