# Supplementary material for: Organizational humanization: definition and measurement
Source: Front Psychol. 2026 Oct 20;17:1906924. doi: 10.3389/fpsyg.2026.1906924 (PMC13727408; doi:10.3389/fpsyg.2026.1906924)
Supplement: Supplementary file 1 [file Table_1.docx]

**Supplementary Table 1**

*Organizational Humanization Scale*

| Item | Statement |
| --- | --- |
| 1 | I am allowed to influence how I do my activities here. |
| 2 | I am allowed to influence when I do my activities here. |
| 3 | I am allowed to influence how my activities are evaluated here. |
| 4 | My uniqueness is valued here. |
| 5 | My uniqueness is embraced here. |
| 6 | My uniqueness can be expressed here. |
| 7 | People show respect in interactions with me here. |
| 8 | People show respect for what I do here. |
| 9 | People show respect to me the same as they do to others here. |

*Note*. Response options are 1 = Strongly disagree, 2 = Disagree, 3 = Neither agree nor disagree, 4 = Agree, 5 = Strongly agree.
